# Analysis of direct and indirect genetic effects in fighting sea anemones

**DOI:** 10.1093/beheco/arz217

**Published:** 2020-01-10

**Authors:** Sarah M Lane, Alastair J Wilson, Mark Briffa

**Affiliations:** 1 School of Biological and Marine Sciences, Animal Behaviour Research Group, University of Plymouth, Plymouth, Devon, UK; 2 Centre for Ecology and Conservation, University of Exeter (Penryn Campus), Cornwall, UK

**Keywords:** *Actinia equina*, aggression, competition, indirect genetic effects, sea anemones

## Abstract

Theoretical models of animal contests such as the Hawk-Dove game predict that variation in fighting behavior will persist due to mixed evolutionarily stable strategies (ESS) under certain conditions. However, the genetic basis for this variation is poorly understood and a mixed ESS for fighting can be interpreted in more than one way. Specifically, we do not know whether variation in aggression within a population arises from among-individual differences in fixed strategy (determined by an individual’s genotype—direct genetic effects [DGEs]), or from within-individual variation in strategy across contests. Furthermore, as suggested by developments of the original Hawk-Dove model, within-individual variation in strategy may be dependent on the phenotype and thus genotype of the opponent (indirect genetic effects—IGEs). Here we test for the effect of DGEs and IGEs during fights in the beadlet sea anemone *Actinia equina*. By exploiting the unusual reproductive system of sea anemones, combined with new molecular data, we investigate the role of both additive (DGE + IGE) and non-additive (DGE × IGE) genetic effects on fighting parameters, the latter of which have been hypothesized but never tested for explicitly. We find evidence for heritable variation in fighting ability and that fight duration increases with relatedness. Fighting success is influenced additively by DGEs and IGEs but we found no evidence for non-additive IGEs. These results indicate that variation in fighting behavior is driven by additive indirect genetic effects (DGE + IGE), and support a core assumption of contest theory that strategies are fixed by DGEs.

## Teaser

We show that for beadlet sea anemones, the likelihood of winning a fight depends on both the genotype of the individual and the genotype of its opponent. We find heritable variation in fighting success and evidence that fights last longer between more related opponents. Our results indicate that an individual’s ability to win will vary from fight to fight depending on the genetic identity of its opponent.

## INTRODUCTION

In many species, the acquisition of resources necessary for survival and reproduction (i.e., mates, territory, and food) is reliant upon engaging in and winning agonistic contests. Thus any trait that increases an individual’s ability to win a contest (resource holding potential—RHP) will enhance its fitness and be subject to positive directional selection. Under such selection, we might naively expect to see among-individual variation in these traits diminish; however, studies of agonistic behavioral traits have found evidence for high levels of among- and within-individual variation (e.g., Wilson et al. 2011; Jennings et al. 2013; Santostefano et al. 2016). This raises the question of how variation in these traits is able to persist in the face of directional selection.

Game-theoretic models have long been used to explore ways in which the balance between the fitness costs and benefits of fighting can lead to maintenance of variation in aggression. The Hawk-Dove game, for example, demonstrates how negative-frequency dependent selection can lead to a stable mix of strategies showing different levels of aggression (Maynard Smith and Parker 1976; Maynard Smith 1982). Under this model, Hawks always fight whereas Doves never do, relying only on displays. Importantly, while Hawks always win contests against Doves, the average fitness pay-off for Hawks encountering other Hawks is diminished by the chance of injury, a feature that is absent when Doves meet Doves. The result is that when the cost of injury is high compared to the value of the resource, variation in fighting strategy will persist, a phenomenon that has been upheld in some animal populations (Tainaka et al. 2007). However, the genetic underpinnings of this variation are poorly understood due to the lack of empirical studies obtaining repeated measures of individual behavior across multiple contests. Consequently, we do not know whether variation in aggression within a population arises from among-individual differences in (fixed) strategy, or from within-individual variation in strategy across contests. In the latter case, any within-individual variation in strategy would be independent of the current opponent. Alternatively, as allowed by subsequent developments of the original model variation in fighting behavior might be conditional on the current opponent (Maynard Smith 1982). For instance, the original model was extended to incorporate a third strategy, “Assessor,” in which individuals assess their opponent, and plastically adjust their own behavior in response to rival phenotype. Thus, evolutionary game theory, suggests that frequency-dependent selection could produce variation in aggressive behavior through three routes; 1) consistent variation among individuals, 2) within-individual variation independent of the opponent, and 3) within-individual variation dependent on the opponent. While phenotypic variation is generally assumed to be dependent on an individual’s genes (direct genetic effects—DGEs) and its biotic and abiotic environment, mounting evidence indicates that a specific biotic component, the social environment, is of particular importance for behavioral traits (Moore et al. 1997; Wolf et al. 1998, 1999; Agrawal et al. 2001; Bijma et al. 2007; Bijma and Wade 2008; McGlothlin et al. 2010; Wilson 2014; Marjanovic et al. 2018). In a general sense, the social environment refers to the conspecifics that an individual encounters. Increasing evidence demonstrates that a vast range of behavioral traits can be affected by the phenotype and thus genotype of an individual’s social partners via indirect genetic effects (IGEs) (e.g., locomotion—Signor et al. 2017; anti-predator behavior—Bleakley and Brodie 2009; Mating behavior—Marie-Orleach et al. 2017). IGEs comprise a heritable component of the environment and can have important evolutionary implications, as different genotypes exert selection pressure on one another, shaping evolutionary trajectories (Bijma et al. 2007; Bijma and Wade 2008; Bijma 2011; Bailey et al. 2018). IGEs can work in conjunction with DGEs in either an additive (G + G) or non-additive (G × G) manner. With additive IGEs, an individual’s phenotype depends on its own genotype and that of its opponent, but the value of the individual’s genotype to itself is constant. For example, individual A’s genotype makes it larger than average and thus A tends to win fights repeatedly. When DGEs and IGEs influence behavior non-additively (i.e., via a genotype-by-genotype interaction effect, G × G), the value of individual A’s phenotype is no longer constant, but changes depending on the genotypes of the individuals that it encounters. In the context of contest behavior, social environmental effects on focal phenotype arise from encounters with the phenotype (e.g., RHP, personality), and so genotype, of the opponent. Therefore, to fully understand variation in, and evolution of, aggressive behavior, we need to take into account both the DGEs of a focal individual’s genotype and the IGEs arising from the genes of both individuals (Moore et al. 2002; Wilson et al. 2009). Moreover, although DGEs and IGEs are not explicitly part of theoretical models of contest behavior, the three scenarios for variation in aggression derived from game theory can also be understood in the context of these direct and indirect genetic effects. For instance, under the basic Hawk-Dove model an individual’s behavior depends on DGEs with IGEs assumed to be absent (i.e., their fighting behavior is not affected by the strategy played by their opponent). Conversely, the fitness pay-off of playing a given strategy in a particular contest depends non-additively on the combination of interacting genotypes (G × G). If a Dove meets a Hawk it gains nothing, but if it meets another Dove, it has a 50% chance of winning. Likewise, a Hawk fighting a Dove will always win without sustaining an injury but a Hawk fighting another Hawk will have a 50% chance of losing and sustaining an injury (Maynard Smith and Parker 1976; Maynard Smith 1982). Furthermore, in the Hawk-Dove-Assessor game, Assessors play Dove against stronger opponents and Hawk against weaker ones. The Hawk-Dove-Assessor model predicts that Assessor should emerge as a pure ESS, but this is only possible if the value of playing Hawk or Dove is conditional on the RHP of the opponent, in other words, if there is an interaction between focal and opponent RHP. Assuming that genotype contributes to RHP this scenario represents a G × G interaction.

Although both additive and non-additive IGEs are implicit in classic game-theoretic models of contest behavior, the extent to which they actually do influence fighting behavior has received little empirical attention to date. Specifically, although we are beginning to understand the importance of additive IGEs in maintaining variation in aggression (Wilson et al. 2009, 2011; Saltz 2013; Alemu et al. 2014; Santostefano et al. 2016; Han et al. 2018), the role of G × G interactions in determining agonistic behavior has yet to be tested empirically. The lack of empirical studies investigating the role of G × G interactions for contest behavior is likely to be largely due to the difficulty in obtaining suitable data from diploid sexual organisms generally used in studies of aggression. In particular, standard variance partitioning approaches require repeated observations not just at the level of each genotype (required for additive DGE + IGE effects), but also at the level of each genotypic combination (required for non-additive DGE × IGE effects). Furthermore, in order to avoid the confounding influence of individual experience, these observations must be carried out on different individuals/ pairs of individuals, meaning that for each genotype, multiple individuals are required. For this reason, investigations into G × G interactions have been restricted to artificial contexts such as inbred lines (Chenoweth et al. 2010; Signor et al. 2017) and in rare cases through the use of clonal organisms (e.g., fungus—Rode et al. 2017).

Many species of sea anemone, for example, are capable of reproducing asexually through somatic embryogenesis, by which a genetically identical individual develops from a single cell and is brooded internally in the coelenteron (gastric cavity) of the parent. The beadlet sea anemone *Actinia equina* has become a model species for studying fighting behavior (Rudin and Briffa 2011, 2012; Lane and Briffa 2017a, 2017b, 2018a, 2018b) and is known to reproduce asexually. Recent evidence has shown that as well as brooding offspring that are genetically identical to themselves, *A. equina* adults tolerate unrelated larvae entering their coelenteron and brood them through the juvenile stage (Lane *et al.* under review). These findings show that *A. equina* broods can be comprised either 1) entirely of clonemates, 2) entirely of genotypically unique individuals or 3) a mix of clonemates and genotypically unique individuals. Moreover, genotypes can differ within broods but be shared between broods, meaning that genetically identical individuals can experience entirely different developmental environments (i.e., non-parental genetically distinct adults). This could have important implications for the expression of traits related to fighting. Specifically, if RHP traits are influenced by properties of the developmental environment (e.g., food supply), genetically identical individuals may express different agonistic phenotypes to a standardized opponent when reared in different broods. Alternatively, if RHP is influenced predominantly by additive or non-additive genetic effects, there may be a high degree of within-brood variation in fighting ability based on genotype.

*Actinia equina* thus provides an ideal model for studying both genotypic and developmental predictors of phenotype, specifically with respect to contest behavior. While previous work has shown that the likelihood of escalation in these fights is significantly influenced by the genetic relatedness between the fighting pair (Foster and Briffa 2014), the role of DGEs and IGEs in determining contest behavior has yet to be explored. Here, we use this model system to investigate the relative importance of DGEs, IGEs (both additive and non-additive) and the developmental environment (i.e., brooding adult identity) on contest outcome and persistence in juvenile *A. equina*. We stage repeated contests at the level of the individual, genotype and at the level of each genotypic combination, using previously developed microsatellite loci to ascertain clonal identity and estimate relatedness between individuals.

## METHODS

### Anemone collection and husbandry

Adult *A. equina* (*n* = 12, yielding *n* *=* 54 juveniles for use in staged contests, see below) were collected from Portwrinkle (Cornwall, United Kingdom; grid reference: SX 357539) between December 2015 and June 2016 and taken back to the lab within 1–2 h of collection. As in previous studies, only anemones of the red/brown color morph were collected as this morph has previously been shown to exhibit higher levels of aggression than anemones of the green/orange morphs found lower down on the shore (Manuel, 1988). Anemones were then individually housed in plastic tanks (23 × 16 cm and 17.5 cm high) containing 700 mL of filtered, aerated seawater and maintained in a controlled temperature room at 15 ± 0.5°C on a 12L:12D lighting cycle. Anemones were fed ad libitum on marine fish flakes every 2–3 days throughout the experiment and seawater was changed every 7 days. Juvenile anemones brooded internally and released in the laboratory by the adults were maintained in the lab at 15 °C in the same tanks as their brood mates and parent until the experiment began. Care was taken not to accidentally exchange juveniles between tanks during water changes.

One week prior to the experiment, juveniles were removed from their brood tanks and isolated in 120 mL pots covered with mesh allowing individual anemones to be identified (*N* = 54 juveniles from 11 broods, average no. juveniles per brood = 4.5). Pots containing brood mates were then placed together in a larger tank (23 × 16 × 17.5 cm) and maintained as described above. Juveniles from different broods were kept in separate tanks.

### Staging contests and sampling tissue

After 1 week of habituation, juveniles from different broods were randomly paired and placed together in a new tank (18 × 11 × 12.5cm). Individuals within pairs were randomly assigned as either the focal or opponent individual and placed such that their body columns were touching. Contests were recorded from this initial contact until either 1) one anemone (the loser) moved a minimum of a pedal disc’s diameter (estimated visually) away from its opponent or 2) retracted all of its tentacles for at least 10 min. (Rudin and Briffa 2011). If both individuals performed these retreating behaviors, the outcome was classified as a draw. Similarly if neither juvenile performed these behaviors, the outcome was classed as a draw. If one or both anemones failed to open their tentacles within 3 h, the interaction was classified as “no fight.” As juvenile *Actinia* (<1 cm in diameter) do not possess acrorhagi (Ottaway 1978), all fights are settled non-injuriously (Rudin and Briffa 2011, Lane and Briffa 2018a), and thus it was not necessary to measure injury or escalation patterns. Fights were recorded using a Canon LEGRIA HF R706 High Definition Camcorder and scored manually for contest duration.

After the fight, juveniles were returned to their individual pots and allowed to recover for 7 days before being introduced to a new opponent and fought again. Each juvenile fought an average of 3.8 times as either the focal or opponent (randomly assigned in each fight), resulting in a total of 86 fights between juveniles from 11 broods.

At the end of the experiment, juvenile anemones were placed in 1.5 mL microcentrifuge tubes containing 100% molecular grade ethanol and frozen at −20 °C for genetic sampling.

### Genotyping individuals

In order to ascertain clonal identity and estimate relatedness between fighting pairs, individuals were genotyped using 8 polymorphic microsatellite markers developed by Ecogenics GmbH (Balgach, Switzerland) as described in Lane et al. (under review).

PCR amplifications were performed in 8 μL reactions containing 182 2 μM of each primer, 5 U of HotStarTaq DNA polymerase (Qiagen, Manchester, United Kingdom), 1 μL 10X PCR buffer (Qiagen, Manchester, United Kingdom), 2 mM of dNTPs (Bioline, London, United Kingdom) and 2.9 μL nuclease-free H2O. A total of 2 μL of 0.5 ng/μL DNA was added to each reaction. Thermocycling was performed on an Alpha Cycler 1 PCRmax thermocycler (PCRmax, Staffordshire, United Kingdom). Cycling conditions consisted of an initial denaturation step of 95°C for 15 min, followed by 35 cycles of 85 °C for 30 s, 56 °C for 45 s, and 72 °C for 45 s, followed by a final step of 72 °C for 30 min. Eight PCR reactions were carried out per sample (one per primer pair).

PCR products were analyzed by Ecogenics GmbH (Balgach, Switzerland) using an ABI3730 (Applied Biosystems) DNA analyzer with an internal size standard (GeneScanTM- 500 LIZ, Applied Biosystems) for accurate sizing. Electropherograms were then visualized using Peak Scanner Software v1.0 (Applied Biosystems) and alleles scored based on amplicon size. The microsatellite sequences used in this paper have been deposited in GenBank (see Supplementary Table S1 for detail of these microsatellites and their accession numbers).

### Genotyping and relatedness

GenAIEx v6.5 (Peakall and Smouse 2006, 2012) was used to calculate the number of multilocus genotypes present and to match individuals by genotype. MLG Sim was then used to calculate an index of genotypic diversity (genotypic diversity = number of genotypes − 1 / number of individuals − 1) (Stenberg et al. 2003; Arnaud-Haond et al. 2007). Genotypic diversity equals zero when a population is dominated by a single genotype and one when all individuals in a population are genetically distinct. Pairwise relatedness was estimated in GenAIEx using the Ritland estimator (Ritland 1996) which is recommended when dealing with highly inbred populations such as those containing clonal individuals (Wang 2017).

### Statistical analyses

Behavioral data were analyzed using a series of linear mixed-effect models fitted using ASReml v4.1. We analyzed three response variables, fight *occurrence* (whether a fight occurred (1) or did not (0)), fight *outcome* scored from the perspective of the designated focal individual (−1 loss, 0 draw, 1 win), and fight *duration* (scored in seconds then log-transformed for analysis). Fight duration data was only available for fights which ended in a clear outcome (i.e., not for fights that ended in a draw). For *outcome* and *duration* we standardized traits to standard deviation units and modeled the data with an assumption of Gaussian residuals. Although they cannot truly be Gaussian, visual inspection of residuals from our model of *outcome* suggests that this approach adequately characterizes the structure of the data. For *occurrence,* we similarly modeled the observed (0,1) data with the same assumption of Gaussian residuals and conducted provisional statistical inference on random effects as described below. While this has the advantage of yielding parameter estimates that are readily interpretable on the observed data scale, the Gaussian assumption is obviously very strongly violated here. We therefore also fitted a parallel series of generalized models for this trait using a logit link and parameter estimation by penalized quasi‐likelihood (PQL) approximation in ASReml (following the approach described for contest outcomes in Wilson et al. 2011).

For each response variable we then fitted a series of models differing in random effect structure following the strategy for modeling dyadic contest data described in Santostefano et al. 2016, 2017, but using clone identity rather than pedigree data to index genotype effects and modeling G × G effects as well. Model 0 was a null model containing a fixed intercept only and no random effects. Model 1 includes random effects of focal and opponent identity only. Since designation of focal versus opponent status is completely arbitrary within the context of each dyadic contest, and only a single (shared) phenotype can be observed it follows that—for *occurrence* and *duration*—the repeatable (additive) effect of any individual *i* on the observation cannot depend on *i*’s designated role. From this, it is necessarily true that V_focal_ = V_opponent_ and the within-individual correlation between focal and opponent effects is +1. The situation is identical for *outcome* in the sense that only one phenotype can be observed per dyad, but the observation recorded now depends on designation. If individual *i* wins a context with *j*, then the outcome is 1 if *i* is the designated focal, but −1 if *j* is the focal. This means that V_focal_ = V_opponent_ as before, but the within-individual correlation between focal and opponent effects is necessarily −1 (see e.g., Wilson et al. 2011 for further explanation). In our models, we, therefore, impose these constraints in order to estimate only those parameters that can be biologically distinct given the trait definitions. This allows us to test for behavioral repeatability and estimate the variance in each observed trait attributable to (additive) effects of the two individual identities.

In Model 2, we then fitted clone identities in addition to individual animal identities. This partitions among individual variance into genetic (clone level) and “permanent environment” (i.e., among-individual, non-genetic) contributions. Random clone effects of focal and opponent were constrained exactly as described for individual effects in Model 1, as were the permanent environmental effects. In Model 3, we added an additional random effect of clone dyad, a factor defined by the interaction of focal clone and opponent clone identities. At least in principle, variance caused by G × G interactions could exist in the absence of additive genetic variance. This scenario would arise in, for instance, a genetically determined paper-rock-scissors game scenario, in which no among-type differences in expected (mean) outcome are predicted (if all types are at equal frequency and interact at random), but all observed variance in contest outcome could be explained by the type combination. Thus, we also fitted one further model (Model 4) that included the G × G interaction and additive effects of individual identity (but not the additive clone identity effects).

Estimated variance components were calculated as a ratio to total phenotypic variance (V_P_) under each model and their significance tested by likelihood ratio tests (LRTs). Specifically, LRTs were used to compare 1) Model 1 against a null model (with no random effects) to test the significance of repeatable (individual-level) variance, 2) Model 2 against Model 1 to test for additive genetic variance in contest behavior and outcome (i.e., DGE + IGE), and 3) Model 3 against Model 2 and Model 4 against Model 1 to test for non-additive (G × G) effects. In each of these comparisons, the more complex model includes a single extra variance component, and we, therefore, conduct LRTs assuming that twice the difference in model log-likelihoods is distributed as a 50:50 mix of $\chi_{0}^{2}$ and $\chi_{1}^{2}$ (Self and Laing 1987). Since the model series is not fully nested we also calculate AIC and use this as a further guide for selecting the preferred model.

Finally, for each trait we refitted the model with the preferred random effect structure (see Results) to test hypothesized fixed effects on mean contest behavior and/or outcome. Specifically, we tested fixed effects of *relative size* (defined as focal-opponent size in SDU of observed size) on *outcome*; and both *size asymmetry* (defined as the absolute value of *relative size*) and microsatellite-based *relatedness* (mean-centered) on *occurrence* and *duration*. Statistical inference on these fixed effects was by conditional F tests implemented in ASReml.

Although our primary focus is on testing for IGEs that might arise from opponent genotype (clone identity) within dyadic contests, in principle effects analogous to IGEs could also arise from the “parental environment” provided by the brooding adults. To check this possibility, we fitted a set of similar models to those described above but using brood identity in place genotype in all random effect structures. Note that genotype is not fully confounded with brood identity here as not all juveniles from a single brood are clonemates (see Lane et al. under review). There were no significant effects of brood identity on any of the response variables and we do not discuss this possibility further (but model detail and results are reported in the Supplementary Material for completeness).

## RESULTS

### Genotypic diversity

Fifteen multi-locus genotypes were identified, which differed by an average of 8.9 alleles out of a possible 16. Of these genotypes, seven were unique (found in only one individual), four were unique to individual broods and the remaining four were shared between broods. The genotypic diversity of the population was 0.37.

### Variation in contest occurrence

Using the models with Gaussian residuals, we found no evidence at all to support additive effects of individual or clonal identity on the binary variable of fight *occurrence*. In fact, corresponding variance estimates were bound at zero with variances constrained to be in allowable (i.e., positive) parameter space (Table 1). Nor was there any evidence for G × G effects, with low effect sizes (5.64% of variance) estimated under Models 3 and 4 that are nominally NS using the LRT (Table 1). AIC scores show the null model (Model 0) is again preferred but we tested fixed effects in a model containing random additive effects of individual identities to protect against pseudo-replication. This provided no evidence that *size asymmetry* (coefficient = −0.049 (0.079), *F*_1,128_ = 0.32, *P* *=* 0.528) or relatedness (coefficient = 0.603 (0.787), *F*_1,116.6_ = 0.59, *P* *=* 0.445) affects whether or not a fight occurs.

**Table 1:** Linear mixed models of contest occurrence, outcome, and duration showing proportions of observed variance (with SE) explained by the random effects as included under each model

Trait	Model	Among-individual (focal + opponent)	Additive genetic (DGE + IGE)	Permanent environment (focal + opponent)	DGE × IGE	LogL	AIC	Comparison	$\chi_{0.1}^{2}$	*P*
*Occurrence*	0					28.874	−55.749			
	1	0.000 0.000				28.874	−53.749	1 vs. 0	0	0.5
	2		0.000 0.000	0.000 0.000		28.874	−51.749	2 vs. 1	0	0.5
	3		0.000 0.000	0.000 0.000	0.056 0.110	28.955	−49.910	3 vs. 2	0.161	0.344
	4	0.000 0.000			0.056 0.110	28.955	−51.910	4 vs. 1	0.161	0.344
*Outcome*	0					−44.724	91.448			
	1	0.406 0.151				−41.495	86.990	1 vs. 0	6.458	0.006
	2		0.401 0.164	0.058 0.111		−37.227	80.455	2 vs. 1	8.535	0.002
	3		0.401 0.164	0.058 0.111	0.000 (-)	−37.227	82.455	3 vs. 2	0	0.5
	4	0.390 0.198			0.147 0.170	−41.441	88.882	4 vs. 1	0.108	0.371
*Duration*	0					−31.549	65.098			
	1	0.151 0.217				−31.299	66.597	1 vs. 0	0.501	0.239
	2		0.229 0.186	0.031 0.192		−30.285	66.570	2 vs. 1	2.027	0.077
	3		0.169 0.198	0.124 0.186	0.253 0.211	−29.611	67.221	3 vs. 2	1.349	0.123
	4	0.198 0.194			0.373 0.197	−30.010	66.019	4 vs. 1	2.578	0.054

Model 0 is a null model with no random effects. In Model 1, focal and opponent identities were fitted whereas Model 2 decomposes these into additive genetic effects and permanent environmental effects. Models 3 and 4 include the non-additive G × G interaction term with (Model 3) or without (Model 4) additive genetic effects. Also shown are model AIC, log-likelihoods and likelihood ratio test comparisons between nested models. All models shown assume Gaussian errors.

While stressing that statistical inference in these models is compromised by the violation of assumed Gaussian residuals, generalized models yielded similar “null” conclusions. Using a logit link, variances for individual, additive, and permanent environment effects were all bound to zero in all models where included. Under Models 3 and 4, the G × G variance (SE) was estimated as 0.419 (0.552) which yields an estimated clone pair “repeatability” of 0.091 on the link scale (i.e., V_G×G_ / (V_G×G_ + V_R_ + π ^2^/3) where π ^2^/3 is the variance on the underlying logit scale). Although formal inference of the G × G effect is slightly problematic using PQL (i.e., LRT are not valid) the ratio of estimated variance to SE provides a useful guide (see Wilson et al. 2011) and here is just 0.76. So clearly, V_G×G_ should not be viewed as statistically significant. Nor does the generalized model provide any support for nominally significant fixed effects in a model including random identity effects only (*asymmetry* coefficient = −0.202 (0.362), *F*_1,128_ = 0.31, *P* *=* 0.573; *relatedness* coefficient = 2.725 (3.640), *F*_1,114_ = 0.56, *P* *=* 0.455).

### Variation in contest outcome

By comparing the log-likelihood value for Model 1 against that of the null model (intercept only), we found evidence of significant among-individual variation in contest outcome, indicating that there are consistent, repeatable differences among individuals in how likely they are to win a fight (as the focal) and in their effects on the fighting success of others (as the opponent). Under Model 1, additive effects of focal and opponent individual identities explained a combined 40.6% (SE = 15.1%) of the variance (i.e., 20.3% attributable to the identity of the focal and the same to the opponent). Including additive genetic (i.e., clone identity) effects significantly improved the model (comparison of Models 1 and 2; Table 1), and suggested most of the among-individual variance was explained by additive genetic effects. Combining across focal and opponent roles, additive genetic effects of clone identity explained 40.1% (SE = 16.4%) of variance in outcome while permanent environment effects accounted for just 5.8% (SE = 11.1%). However, the model was not further improved by the inclusion of the G × G effect (comparison of Model 3 to 2; Table 1) and in fact, this variance component was bound to zero in Model 3. Nor did the comparison of Models 4 and 1 provide evidence of G × G. Thus we find evidence of additive genetic effects (DGE + IGE) but not of non-additive G × G. Refitting Model 2 with an additional fixed effect of relative size demonstrated no support for body size being an important determinant of RHP (relative size effect; SE = 0.095 (0.132), *F*_1_,_84_ = 0.52, *P* *=* 0.470).

### Variation in contest duration

Under Model 1, we estimated additive effects of focal and opponent individual identities, which explained a combined 15.1% (SE = 21.7%) of variance in (log-transformed) contest duration. This among-individual variance was not statistically significant (comparison of Model 1 to Model 0; Table 1). Extension to Model 2 yielded estimates of 22.9% (SE = 18.6%) and 3.1% (SE = 19.2%) of observed variance in contest duration attributable to additive genetic and permanent environment effects, respectively. Unsurprisingly given the lack of evidence for individual identity effects, there was no statistical support for significant additive genetic effects (Table 1). Nor indeed was there evidence of significant non-additive effects (see comparisons of Model 3 vs. 2 and 4 vs. 1 in Table 1) though the corresponding estimated effect sizes were large (e.g., G × G accounting for 25.3% and 37.3% of variance in Models 3 and 4, respectively).

**Figure 1:**
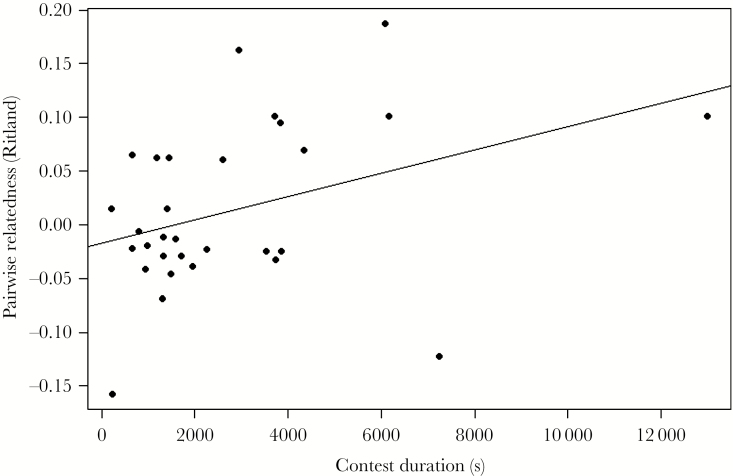
Effect of pairwise relatedness (Ritland estimator) on contest duration.

While no random effects were statistically significant and the null model actually had the lowest AIC, we tested for effects of size asymmetry and relatedness by adding these as fixed effects to a model containing random identity effects as specified in Model 1. This was to prevent pseudo-replicating for inference on the fixed effects. The effect of size asymmetry was negative as predicted, though marginally nonsignificant (coefficient = −0.419 (0.218), *F*_1,57_ = 3.69, *P* *=* 0.060) Thus fights between more evenly sized individuals tend to be of longer duration. There was, however, a significant positive effect of relatedness on fight duration (coefficient = 5.676 (2.259), *F*_1,57_ = 6.31, *P* *=* 0.015). Thus, as the relatedness of opponents increased, so did the duration of the contest (Figure 1).

## DISCUSSION

Agonistic contests involve two or more interacting individuals, and thus, the aggressive phenotypes expressed during contests may be dependent not only on an individual’s genotype (DGEs) but also on the genotype of its opponent (IGEs). In this study, we investigated the role of IGEs in determining the contest behavior of juvenile beadlet anemones *A. equina*. Our analysis showed that among-individual variation in fighting success was largely explained by additive DGEs and IGEs (G + G). Thus, the data support an underlying assumption implicit in models such as the hawk-dove game that selection acts on strategies fixed by DGE. In contrast, we found only limited evidence for non-additive G × G interactions (on contest duration) and no evidence of a genetic influence at all on the likelihood of fights occurring. Fight duration did not appear to be explained by direct or indirect genetic effects, and so is not a heritable trait per se. Nonetheless, there was a significant positive relationship between contest duration and relatedness, with fights continuing for longer as the relatedness between two opponents increased. Thus the expressed phenotype is actually contingent on the particular combination of genotypes involved in the dyad, but not because of heritable variation in RHP (which would manifest as variance attributable to DGE and IGE).

Over 40% of among-individual variation in fight outcome was explained by additive direct and indirect genetic effects. This means that an individual’s chance of winning a fight was determined by an (additive) combination of its own genotype, and its opponent’s genotype affecting RHP. Although our results indicate that fighting behavior does not differ significantly between broods, because juvenile genotype varies within broods in *A. equina*, and fighting success is in part determined by DGEs, fighting ability (RHP) is likely to vary within broods. Furthermore, as we found no effect of developmental environment (brood ID) on fighting success, this variation in RHP should persist regardless of the identity (and perhaps genotype) of the brooding adult. This is particularly interesting for broods which contain a mix of clonemates and unique individuals (i.e., in which some of the juveniles are genetically identical to the brooding adult and some are not). *Actinia equina* fight over the acquisition of territory on the intertidal zone, requiring a sheltered spot to avoid desiccation, and thus possible death, when the tide goes out. Depending on the genotype of the brooding adult and on the genotypes of fostered juveniles, it is possible that the adult’s own genetically identical progeny may be outcompeted by their foreign brood mates. This possibility could be detrimental to the brooding adult’s fitness and thus raises the question as to why adults tolerate foreign juveniles within their coelenteron. One explanation is that adults are unable to distinguish between their own progeny and unrelated young. Although it is generally accepted that adult anemones are able to identify self from non-self (Bigger 1980; Lubbock 1980; Turner et al. 2003), very little is known about whether adults can distinguish between their own young and non-related juveniles (although see: Lubbock and Allbut 1981; Ayre 1982), especially at the larval stage when it is thought foreign offspring first enter the coelenteron.

We found no evidence for the presence of non-additive genetic effects (G × G) for any of the fight parameters measured. As discussed above, detection of G × G interactions requires adequate replication at the level of the genotype and the level of each genotypic combination. As we were unable to genotype individuals prior to engaging in fights (tissue sampling in juvenile anemones is fatal), we could not ascertain what the number of replicates would be at each level until after the experiment (an average of 8.29 fights per genotype and 1.75 per genotypic combination). However, if the lack of evidence for G × G were due to power limitations, we might expect the variance explained by G × G to still be substantial, albeit nonsignificant. However, the variance components relating to G × G in our analysis were either bound or very close to zero in all models. Thus, even if our replicate size resulted in power limitations, G × Gs are unlikely to be of biological importance for *A. equina*. This finding also reflects the persistence of linear dominance hierarchies in natural populations. If non-additive genetic effects (G × Gs) were of widespread importance in determining fighting behavior we would expect to see the formation of genetically determined intransitive dominance hierarchies in natural populations (i.e., hierarchies in which the value of an individual’s genotype is dependent on the genotype of its opponent, such that just because *A* beats *B* and *B* beats *C*, we cannot assume that *A* will be dominant to *C*), but comparative studies have shown transitive dominance hierarchies to be prevalent and intransitive hierarchies to be rare (Chase et al. 2002; McDonald and Shizuka 2012). Thus the presence of G + G and absence of G × G interactions in *A. equina* is consistent with this common pattern of linear transitive hierarchies and may reflect a general trend found across diverse study systems.

In contrast to the results on fighting success, we found no among-individual variation for contest occurrence or duration (i.e., persistence) nor any significant influence of direct or indirect genetic effects on these parameters. Although selection is expected to favor any trait that increases the likelihood of victory, fighting success is not determined by persistence alone but rather by a functionally diverse suite of traits (Kemp et al. 2006; Vieira and Peixoto 2013; Wilson et al. 2013). Furthermore, at present, the genetic architecture of RHP traits (e.g., body size, weaponry, personality) has yet to be elucidated. Thus detecting genetic effects on a single contributing trait such as persistence is likely to be more difficult than detecting genetic effects on fighting success itself. Furthermore, persistence is not only reliant upon underlying RHP traits, but is also affected by an individual’s perception of resource value (Enquist and Leimar 1987; Kemp et al. 2006; Palaoro et al. 2017), a factor known to be sensitive to both intrinsic and environmental conditions (Stockermans and Hardy 2013; Lane and Briffa 2018b). Genetic effects on a parameter determined by such plastic responses may thus be difficult to detect.

Despite not finding any direct or indirect genetic effects on contest duration, we did find a significant positive relationship between contest duration and relatedness, indicating that fights lasted longer between more related individuals. This finding is contrary to general predictions concerning relatedness and aggression but is consistent with a study of adult *A. equina* in which fights were found to escalate more often as the relatedness between opponents increased (Foster and Briffa 2014). As juvenile anemones do not possess acrorhagi, they are unable to escalate to injurious fighting; instead, they must simply persist longer than their opponent if they are to win. Our results also indicated a marginally nonsignificant trend between contest duration and size asymmetry, suggesting that fights were longer between more similarly matched individuals. As opponents become more similar in terms of their RHP, theory predicts that fights will be harder to settle and thus, fights are expected to go on for longer and be more likely to escalate. Foster and Briffa (2014) suggested that relatedness may covary with the expression of RHP traits in *A. equina*, meaning that more related individuals will be more closely matched in terms of RHP. Although we found no evidence of a relationship between size asymmetry and relatedness in our study, suggesting these two factors influence contest duration separately, it is possible that other RHP traits such as weaponry or boldness (Rudin and Briffa 2012) covary with relatedness in this species.

In summary, here we provide direct evidence that fighting behavior is dependent on both direct and indirect genetic effects. We show that an individual’s success in any particular fight is dependent on an additive combination of its own genetic value for RHP and that of its opponent. However, we find no evidence of G × G interactions, which—if present—would suggest the possibility of non-linear dominance hierarchies emerging. Nor do we find evidence for DGE or IGE on fight occurrence or duration (given a fight does occur), although the latter is dependent on a genotype-by-genotype interaction in the sense that it increases with pairwise relatedness. Our results thus demonstrate that persistence and success in sea anemone contests are determined by the genotypes of both opponents and that contest success, but not persistence demonstrates heritable variation. Taken together, these effects on contest outcomes validate underlying assumptions implicit in contest theory (ESS solutions depend on the presence of strategies fixed by DGEs) and explanations for the widely observed presence of linear and transitive dominance hierarchies (which require both DGE and IGE, but would be absent with G × G) in animal societies.

## Supplementary Material

arz217_suppl_Supplement_Material
