# Supplementary material for: Analysis of direct and indirect genetic effects in fighting sea anemones
Source: Behav Ecol. 2020 Jan 10;31(2):540–7. doi: 10.1093/beheco/arz217 (PMC7083097; doi:10.1093/beheco/arz217)
Supplement: arz217_suppl_Supplement_Material [file arz217_suppl_supplement_material.docx]

**SUPPLEMENTARY MATERIAL:**

ANALYSIS OF DIRECT AND INDIRECT GENETIC EFFECTS IN FIGHTING SEA ANEMONES

Sarah M. Lane^*^, Alastair J. Wilson & Mark Briffa

* corresponding email: [sarah.lane@plymouth.ac.uk](mailto:sarah.lane@plymouth.ac.uk)

**Table S1** Characteristics of the nine *Actinia equina* microsatellite markers used in this study. Shown are the number of alleles (A), alongside observed (H_o_) and expected (H_e_) heterozygotes per locus; p-value and associated standard error (SE) for heterozygosity deficiency per locus.

|  |  |  |  |  |  |  | Het Deficit | |  |
| --- | --- | --- | --- | --- | --- | --- | --- | --- | --- |
| Locus | Repeat motif | Primer sequences 5' - 3' | Size range (bp) | A | H_o_ | H_e_ | *P* value | SE | Acc. No |
| 1 | (ATGG)_23_ | F: GAATCGGTCACTGGCCTTG R: ATAAAACTCAAGGCAGAATTAGTTAAG | 188-278 | 8 | 51 | 57 | 0.1571 | 0.112 | Ae_38886 |
| 2 | (ATA)_14_ | F: AGCGCCTTGGACAATTTTGG R: TTTGGTCGTCGAACTCCCAG | 170-216 | 8 | 80 | 65 | 1 | <0.0001 | Ae_158678 |
| 3 | (TA)_13_ | F: AGCGTGGATGACAGCGTTAG R: ATGCGTCGACCAATAGGAGG | 220-300 | 5 | 36 | 40 | 0.2796 | 0.0117 | Ae_209988 |
| 4 | (AAGG)_12_ | F: TGCCCATCGAATGTAAGCAAC R: AACTTAGGTTCAAATACGACCG | 156-192 | 9 | 71 | 69 | 0.917 | 0.0111 | Ae_366260 |
| 5 | (AGAC)_7_ | F: GTACGTCAAGTCGTTCTGCC R: TTACTGATGAGACGTCCGTG | 208-260 | 5 | 36 | 34 | 0.9294 | 0.065 | Ae_383411 |
| 6 | (TA)_15_ | F: TCGAAGCTGCCAAAACATCG R: CGTGCTAATCTTACCAAAAGTGTG | 193-235 | 8 | 48 | 63 | <0.0001 | <0.0001 | Ae_425626 |
| 7 | (CTA)_30_ | F: TCTATGGATACCCAACTTTAAGGC R: TGCACCAATACCAGGTTCCC | 176-200 | 9 | 74 | 67 | 0.96 | 0.008 | Ae_545782 |
| 8 | (TC)_16_ | F: TGGCCAAATTAACCTTTTCCG R: ACAGTGTTGGAAATAGGCCG | 135-145 | 4 | 55 | 56 | 0.67 | 0.01 | Ae_573755 |
| 9 | (ATT)_13_ | F: GGTTTGACTTTGCTTTTTCATTGG R: AGAGCCAACATACCTCCGAC | 199-217 | 5 | 42 | 42 | 0.06 | 0.006 | Ae_696810 |

**Table S2** Linear mixed models of contest occurrence, outcome and duration showing proportions of observed variance (with SE) explained by the random effects as included under each model. Model 0 is a null model with no random effects In model 1, focal and opponent identities were fitted while Model 2B decomposes these into additive brood identity effects and permanent environment effects. Models 3B and 4B include the non-additive brood x brood interaction term with (Model 3B) or without (Model 4B) additive brood identity effects. Also shown are model AIC, log-likelihoods and likelihood ratio test comparisons between nested models. All models shown assume Gaussian errors.

| Trait | Model | Among-individual (focal + opponent) | Additive brood  (Focal + Opponent) | Permanent environment (focal +opponent) | Brood_f_ x Brood_o_ | LogL | AIC | Comparison | Χ^2^_0.1_ | P |
| --- | --- | --- | --- | --- | --- | --- | --- | --- | --- | --- |
| *Occurrence* | 0 |  |  |  |  | 28.874 | 28.874 |  |  |  |
|  | 1 | 0.000 0.000 |  |  |  | 28.874 | 28.874 | 1 vs 0 | 0 | 0.5 |
|  | 2B |  | 0.000 0.000 | 0.000 0.000 |  | 28.874 | 28.874 | 2 vs 1 | 0 | 0.5 |
|  | 3B |  | 0.000 0.000 | 0.000 0.000 | 0.143 0.122 | 29.636 | 29.636 | 3 vs 2 | 1.524 | 0.108 |
|  | 4B | 0.000 0.000 |  |  | 0.143 0.122 | 29.636 | 29.636 | 4 vs 1 | 1.524 | 0.108 |
| *Outcome* | 0 |  |  |  |  | -44.724 | 91.448 |  |  |  |
|  | 1 | 0.406 0.151 |  |  |  | -41.495 | 86.990 | 1 vs 0 | 6.458 | 0.006 |
|  | 2B |  | 0.070 0.114 | 0.321 0.180 |  | -41.312 | 88.624 | 2 vs 1 | 0.366 | 0.273 |
|  | 3B |  | 0.068 0.119 | 0.312 0.175 | 0.120 0.152 | -40.991 | 89.982 | 3 vs 2 | 0.642 | 0.212 |
|  | 4B | 0.3854 0.1527 |  |  | 0.1314 0.1532 | -41.1487 | 88.2974 | 4 vs 1 | 0.6926 | 0.202641 |
| *Duration* | 0 |  |  |  |  | -31.549 | 65.098 |  |  |  |
|  | 1 | 0.1506 0.2174 |  |  |  | -31.2986 | 66.5972 | 1 vs 0 | 0.5008 | 0.239574 |
|  | 2B |  | 0.1166 0.1433 | 0.0778 0.2184 |  | -30.8735 | 67.747 | 2 vs 1 | 0.8502 | 0.178248 |
|  | 3B |  | 0.1166 0.1433 | 0.0778 0.2184 | 0.000 (-) | -30.8735 | 69.747 | 3 vs 2 | 0 | 0.5 |
|  | 4B | 0.1506 0.2174 |  |  | 0.000 (-) | -31.2986 | 68.5972 | 4 vs 1 | 0 | 0.5 |
